# Functionalized self-assembled monolayers on mesoporous silica nanoparticles with high surface coverage

**DOI:** 10.1186/1556-276X-7-334

**Published:** 2012-06-21

**Authors:** Liangming Wei, Diwen Shi, Zhihua Zhou, Peiyi Ye, Jian Wang, Jiang Zhao, Liyue Liu, Changxin Chen, Yafei Zhang

**Affiliations:** 1 Key Laboratory for Thin Film and Microfabrication of Ministry of Education, Institute of Micro and Nano Science and Technology, Shanghai Jiao Tong University, Shanghai, 200240, China; 2 State Key Laboratory of Electronic Thin Film and Integrated Devices, School of Microelectronics and Solid-state Electronics, University of Eectronic Science and Technology of China, Chengdu, 610054, China; 3 School of Materials Science and Engineering, Shanghai Jiao Tong University, Shanghai, 200240, China; 4 School of Chemistry and Chemical Engineering, Shanghai Jiao Tong University, Shanghai, 200240, China

## Abstract

Mesoporous silica nanoparticles (MSNs) containing vinyl-, propyl-, isobutyl- and phenyl functionalized monolayers were reported. These functionalized MSNs were prepared via molecular self-assembly of organosilanes on the mesoporous supports. The relative surface coverage of the organic monolayers can reach up to 100% (about 5.06 silanes/nm^2^). These monolayer functionalize MSNs were analyzed by a number of techniques including transmission electron microscope, fourier transform infrared spectroscopy, X-ray diffraction pattern, cross-polarized Si^29^ MAS NMR spectroscopy, and nitrogen sorption measurement. The main elements (i.e., the number of absorbed water, the reactivity of organosilanes, and the stereochemistry of organosilane) that greatly affected the surface coverage and the quality of the organic functionalized monolayers on MSNs were fully discussed. The results show that the proper amount of physically absorbed water, the use of high active trichlorosilanes, and the functional groups with less steric hindrance are essential to generate MSNs with high surface coverage of monolayers.

## Background

Periodic mesoporous silica materials have been intensively investigated over the last decade due to their ordered structure, large surface area, and well-defined pore size [1,2]. Mesoporous silica materials with different morphologies, such as spheres [3], fibers [4], films [5], and vesicles [6] have been developed. Very recently, small mesoporous silica nanoparticles (MSNs) have been receiving much attention [7-14]. Compared with bulk mesoporous silica (typical diameter of 500 nm to several micrometers), MSNs (diameters ranging from 50 to 500 nm) offer additional properties, such as fast mass transfer of molecules into or out of the pore systems, effective adhesion to the substrates, good suspension in solution, and easy permeability across cell membrane [15]. These features make MSNs excellent candidates for drug delivery vehicles [10], sensors [16], and catalyst supporter [17]. However, many applications require these materials to have special binding sites, stereochemical configuration, charge density or surface, and interface properties (such as wetting and adhesion) [18-20]. So, it is necessary to develop methods to modification of the large surface by organic functional groups to tailor the surface properties or to bind special targets, thus extending the use of MSNs to wide application [21,22].

So far, two main pathways are available for functionalization of MSNs with organic groups. One is the grafting method based on direct reaction between organosilane and surface silanols [23,24]. The limitation of this synthetic method is that the population of the organic groups would be limited to the original number of surface silanols on MSNs, and thus, a low surface coverage is obtained (<25%) [23-25]. Another approach for functionalization of MSNs is the one-pot synthesis method through co-condensation of tetraalkoxysilanes with terminal trialkoxyorganosilanes in the presence of structure-directing agents, leading to materials with organic residues anchored covalently to the pore walls [18,21]. Using this method, the organic MSNs with narrow particle size distribution in the range of about 40 to 150 nm could be synthesized,^21^ but this method leads to disordered MSNs, and the content of organic functionalities in the modified silica phases does not normally exceed 40 mol% [18,21].

**Figure 1:**
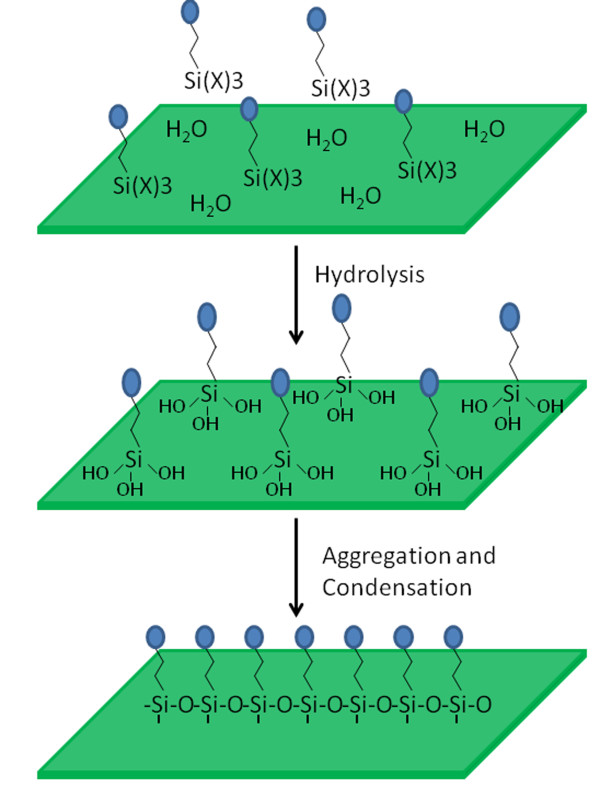
**The schematic illustration of self-assembly of organosilanes on the ceramic oxide surface.** Hydrolysis of organosilanes on the oxide surfaces to create the corresponding hydroxylsilanes, and then, these hydroxylsilanes absorb on the surface via hydrogen bond. Aggregation and condensation of these hydrogen-bound species lead to a closely packed monolayers on the oxide surface.

Molecular self-assembly of organosilanes has been proven to be a powerful method to rationally engineer the ceramic oxide surface properties [26-29]. In this process, the organo-silanes are hydrolyzed to create the corresponding hydroxylsilanes on the polar oxide surfaces, and then, these hydroxylsilanes absorb on the surface via hydrogen bond. Aggregation of these hydrogen-bound species, which driven by the attractive van der Waal^’^s force between the pendant hydrocarbon chain and cross-linking between neighboring silanes, as well as condensation between the hydroxylsilanes and the polar oxide surface lead to a closely packed monolayers on the oxide surface (Figure 1) [23]. Using this method, the relative surface coverage of the monolayers can reach 100% [29]. So far, this method has been successfully applied for functionalizing the bulk mesoporous silicas [20,30,31], but application of this method to produce the monolayer functionalized MSNs with high coverage failed. One possible reason is that the functional molecules are very easy transferred out of the pores of MSNs during functionalization process, leading to poor surface coverage. 

Here, we extent the self-assembled method toward the generation of self-assembled monolayer functionalize MSNs (SAM-MSNs) and show that the surface coverage of the monolayers can be varied up to 100% within MSNs, as well as retention of ordered structure. As described in the following, various organic monolayers could be assembled onto the porous walls of MSNs using this general method.

## Methods

The organosilanes (vinyl-trichlorsilan, propyl-trichlorsilan, isobutyl-trichlorosilane, and phenyl-trichlorsilan) were purchased from Sigma-Aldrich Corporation, St. Louis, MO, USA. Other reagents were purchased from Shanghai Zhenxin Reagents Factory, Jiading, Shanghai. Toluene was dried over 10 Å molecular sieves for 10 days and then refluxed over Na for 10 h. Ultrapure water (σ = 18.2 MΩ cm) was degassed and decarbonated with argon.

Surface analysis of samples was performed on a Micromeritics ASAP 2010 analyzer (GA, USA) at the temperature of liquid nitrogen. The surface areas were calculated by the Brunauer-Emmett-Teller (BET) and pore size distribution was measured by Barrett-Joyner-Halenda (BJH) methods. Samples were outgassed at 100°C for 12 h before analysis. The pore volume was taken at a relative pressure of 0.99. Cross-polarized solid state ^29^Si NMR experiments were recorded on Bruker DSX 300 NMR (Bruker Optik Gmbh, Ettlingen, Germany). The following experimental parameters were used: 4.0 KHz spinning frequency, 45^o^ pulse width of 2.4 ls, 30 s recycle delay, and 360 scans. Fourier transform infrared spectroscopy (FTIR) spectra were obtained using a Bruker Vertex70 spectrophotometer using KBr pellets. The transmission electron microscope (TEM) images were obtained from JEOL 2100 operating at 200 kV (Akishima, Tokyo, Japan). The samples were prepared by evaporation of a drop of sample solution on a grid at room temperature.

For preparation of organic monolayer functionalized MSNs, MSNs were first prepared according to a reported method [32,33]. Typically, 1 g of cetyltrimethylammonium bromide (CTAB) was dissolved in a solution of 480 mL water and 3.5 mL of 2 M NaOH. Mesitylene (7.0 mL) was then added to the solution. The mixture was stirred vigorously at 80°C for 2 h, following which tetraethyl orthosilicate (TEOS) (5.0 mL) was added dropwise. The reaction mixture was stirred for further 2 h at 80°C. The resulting precipitate was collected by filtration, washed with methanol, and dried under a vacuum at 100°C for 24 h. The surfactant templates and mesitylene were extracted from the samples using HCl/methanol solution. A suspension of 1.0 g of the as-synthesized MSNs was stirred for 6 h at 50°C in 100 mL methanol with 0.75 mL concentrated hydrochloric acid. The template-removed product was then collected via filtration and dried under vacuum at100°C for 12 h.

The procedure for functionalization of MSNs with organic monolayers was described as follows: 1 g of MSNs (1095 m^2^ of surface area) was suspended in 50 mL of dried toluene. To this mixture was added 0.33 mL of water (18.22 mmol, corresponding to 1 × 10^19^ water molecules/m^2^, just equal to twice the number of silanol groups present on a fully hydroxylated silica surface).^19^ The suspension was stirred vigorously for 3 h at room temperature to allow the water to disperse through the mesoporous matrix. Then organic trichlorsilans (9.11 mmol, corresponding to 5 × 10^18^ water molecules/m^2^ just equal to the number of silanol groups present on a fully hydroxylated silica surface) were added via syringes, and the mixture was stirred for 12 h at room temperature under N_2_. The produces were collected by filtration, washed with plenty of 2-propanol, and dried under a vacuum.

## Results and discussion

**Figure 2:**
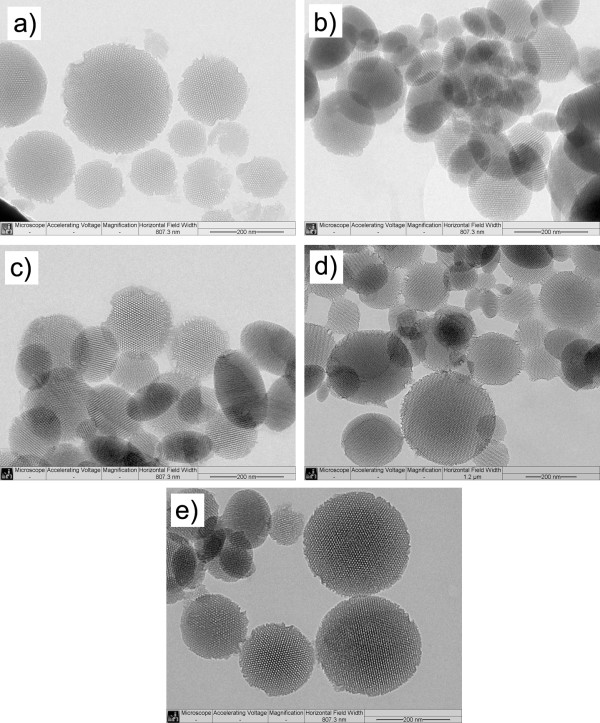
The TEM of MSN (a), Vi-SAM-MSNs (b), Pr-SAM-MSNs (c), Is-SAM-MSNs (d), and Ph-SAM-MSNs (e).

To synthesize the SAM-MSNs, the MSNs were first prepared. MSNs were synthesized in CTAB/OH, TEOS, and mesitylene solution, which have been reported previously [32,33]. As shown in the Figure 2, MSNs have a particle diameter of 100 to 200 nm and composed of ordered hexagonal array of mesoporous channels. The nitrogen surface sorption analysis of MSNs gave the BET surface area of 1,095 m^2^/g^−1^ and the pore size of 6.5 nm. 

SAM-MSNs were synthesized via deposition of organic monolyers on the pore walls of MSNs. First, the surface of MSNs was prehydrated with two monolayers worth of water, followed by treatment with one monolayer worth of organosilanes (based on the available surface area). The organosilanes (vinyl-trichlorsilan, propyl-trichlorsilan, isobutyl-trichlorsilan, and phenyl-trichlorsilan) then underwent hydrolysis and became covalently attached to the substrate and cross-linked to one another, generating SAM-MSNs with different organic groups (denoted Vi-SAM-MSNs, Pr-SAM-MSNs, Is-SAM-MSNs, and Ph-SAM-MSNs, respectively).

The surface coverage of the functional groups could be estimated by gravimetric analysis [20,31]. Based on the weight change (W%) after functionalization, the surface area (SA, 1095 m^2^/g), the molecular weight (M_n_) of the functional groups [OH-Si-(O)-R], and the assumption of 5 × 10^18^ silanols per square meter on a fully hydroxylated silica surface [20,31], surface coverage (*X*) can be calculated from

(1) $$X=\left[\left(W\%/M_{n}\right)\times 6.02\times 10^{23}\right]/\left(SA\times 5\times 10^{18}\right)$$

This gravimetric method of estimating surface coverage is felt to be accurate within ±10% [31]. It should be mentioned that when this gravimetric method was used to determine the surface coverage, the byproducts (e.g., residual polysiloxanes) trapped within the nano-pores should be excluded. Since only two monolayers worth of water and one monolayer worth of organosilanes (based on the available surface area) were used in preparation of SAM-MSNs, few byproducts within the pores could be expected. This assumption was also confirmed by TEM and BET results, which will be discussed below. The surface coverage could also be calculated from solid state ^29^Si NMR spectroscopy, but this method is less accurate than the gravimetric method because of the signal-to-noise limitation of the solid state ^29^Si NMR spectroscopy (accurate within ±15%) [20,31]. Table 1 indicated that the surface coverage was varied from 37% to 100% depending on different organosilanes used. 

**Table 1:** Properties of different self-assembled monolayer functionalize mesoporous silica nanoparticles (SAM-MAN)

**Sample**	**Functional molecules**	**Weight changes after functionalization**	**Coverage (%)**	**BET surface area (m**^**2**^**/g)**	**BJH pore size (nm)**	**Pore volume (cm**^**3**^**/g)**
MSN		0	0	1,095	6.5	2.0
Vi-SAM-MSN	vinyl-trichlorsilan	81	101	438	4.79	0.5
Pr-SAM-MSN	propyl-trichlorsilan	62	65	489	4.63	0.63
Is-SAM-MSN	isobutyl-MSN	40	37	582	5.31	0.94
Ph-SAM-MSN	phenyl-trichlorsilan	100	80	480	4.71	0.56

**Figure 3:**
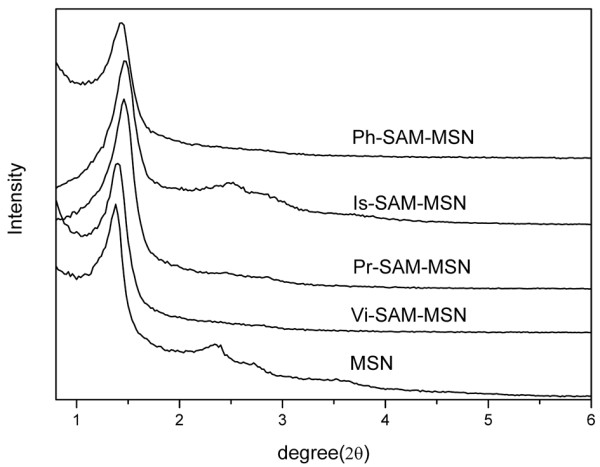
The XRD spectra of MSN and different SAM-MSNs.

Figure 2 shows the TEM of MSNs and the SAM-MSN samples with different functional groups. It could be found that both MSNs and SAM-MSN samples were comprised of MCM-41-type, hexagonal array of mesoporous channels, suggesting the functionalization of MSNs with organic monolayer did not destruct the order hexagonal array of mesoporous channels. From TEM pictures, no free non-porous silica particles were observed in all SAM-MSN samples. This result confirmed that all organic functional groups were anchored on the surface of MSNs. The TEM images also show that the pores are still open even though the 100% of full surface was covered by functional groups (Figure 2b). The powder X-ray diffraction pattern (XRD) of SAM-MSNs further confirmed the retention of the hexagonal symmetry of the porous structure (Figure 3) after functionalization, as evidenced by the distinct *d*_001_ peak in all SAM-MSN samples. However, compared with the XRD of pure MSN sample, a lower contrast of XRD and a slight decrease in the *d*_001_ values were observed. This change in the XRD could be attributed to the covalent linkage of organic groups onto the pore walls [34,35]. 

**Figure 4:**
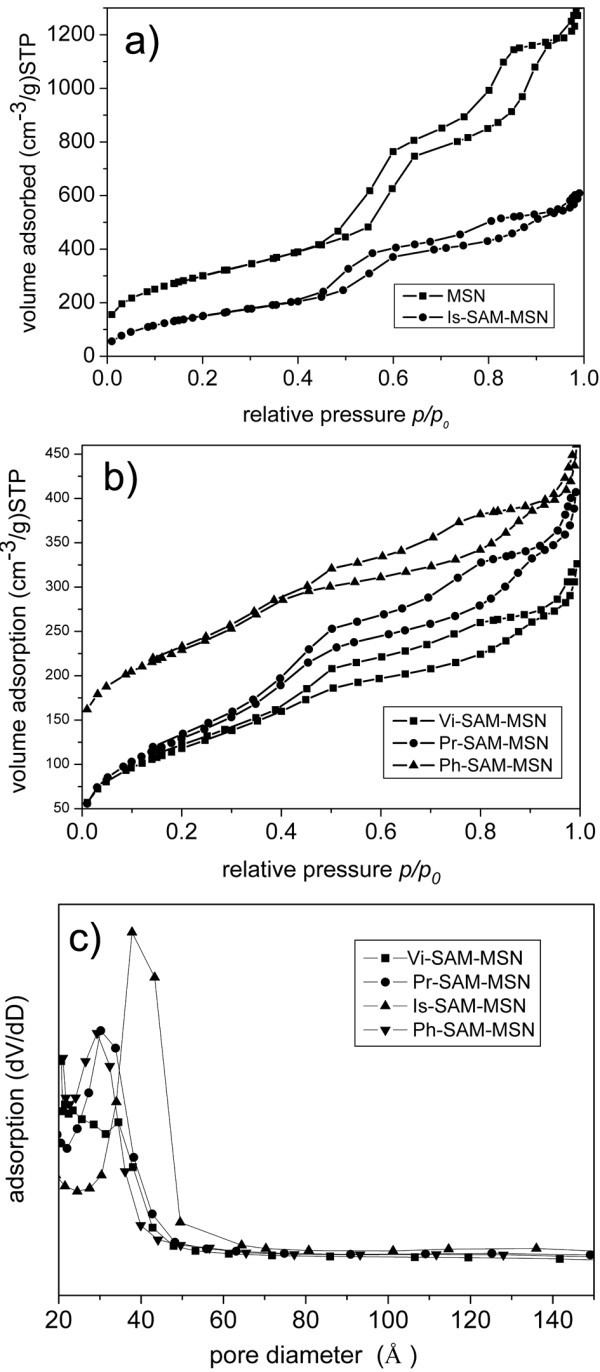
Nitrogen sorption isotherm (a and b) and BJH pore size distribution of different SAM-MSNs.

**Figure 5:**
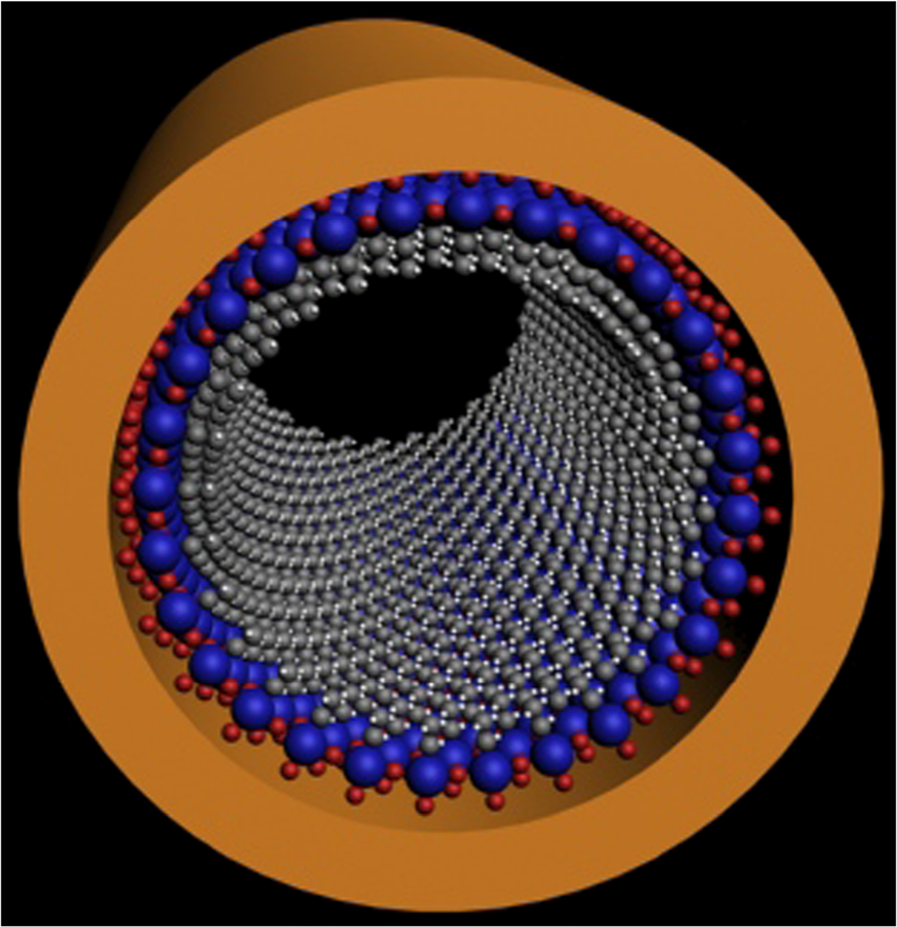
**Schematic drawing of the self-assembled monolayer structure within a mesopore.** The red, blue, grew, and white spheres indicate the oxygen, silicon, carbon, and hydrogen atom, respectively.

The nitrogen adsorption-desorption isotherms and BJH pore size distributions for MSNs and SAM-MSNs are shown in Figure 4, the parameters of which are listed in Table 1. The BET surface area of the functionalized MSNs was about half the value of the pure MSN. The decrease in the surface areas is reasonable because the mass of MSNs increased significantly (by 60% to approximately 100%) after functionalization. The functionalization of MSNs also results in the reduction of pore volume and shrinkage of the BJH pore diameter (Table 1), which was ascribed to the lining of pore wall with the organic functional groups. The BET characterization shows that all SAM-MSNs show type IV sorption isotherms. These isotherm characterizations are consistent with the fact that the functional groups were grafted onto the pore walls, and few free polysiloxanes were trapped in the pores (Figure 5) [36,37]. 

**Figure 6:**
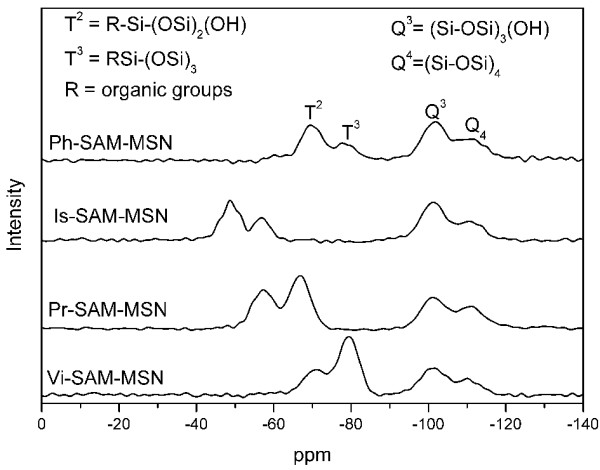
^**29**^**Si -MAS NMR spectra of the different SAM-MSNs.**

**Figure 7:**
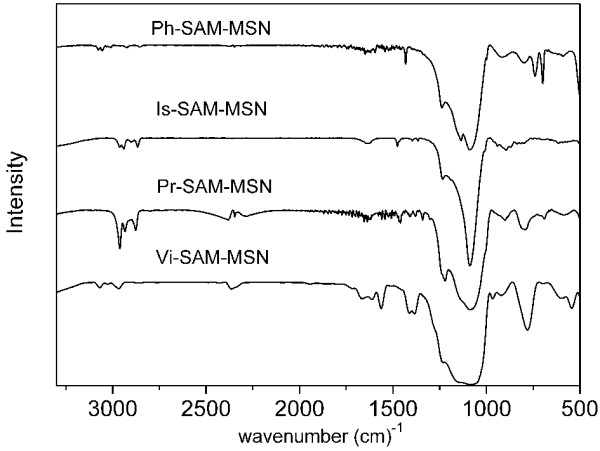
FTIR of different template-extracted SAM-MSM samples.

To confirm the grafting of the organosiloxanes onto the pore walls of MSNs, the samples were analyzed by solid state ^29^Si NMR and FTIR. The ^29^Si NMR spectra (Figure 6 and Table 2) show the presence of two additional peaks, T^2^ and T^3^ [T^2^ = R-Si-(OSi)_2_(OH), T_3_ = RSi-(OSi)_3_, R = organic groups] in the functionalized MSNs. These peaks are ascribed to the resonances of the incorporated organosiloxanes in the functionalized MSNs. FITR spectra further confirmed the functional groups grafted onto MSNs (Figure 7). The peaks around 2,800 to 2,970 cm^−1^, which were assigned to the aliphatic C-H vibrations, were observed in the Pr- (2,960, 2,930, 2,873 cm^−1^) and Is- (2,963, 2,939, 2,866 cm^−1^) SAM-MAN samples. The alkene C-H vibration (3,067 cm^−1^) and C = C stretching vibration (1,570, 1,607 cm^−1^) were observed in the Vi-SAM-MSN sample. The aromatic C-H (3,075, 3,055 cm − ^1^) and ring C = C (1,590, 1,432 cm^-1^) stretching vibrations were clearly observed in Ph-SAM-MSN sample.

**Table 2:** **Cross-polarized solid state**^**29**^**Si-MAS NMR spectroscopy data of SAM- MSNs**

**Sample chemical shift (ppm)**				
	Q4	Q3	T3	T2
Vi-SAM-MSN	Q4	Q3	T3	T2
Pr-SAM-MSN	−110	−101	−79	−70
Is-SAM-MSN	−110	−101	−66	−57
Ph-SAM-MSN	−110	−101	−57	−48

The surface coverage and the quality of the organic functionalized monolayers on MSNs are greatly affected by three facts: the number of absorbed water, reactivity of organosilanes, and the stereochemistry of organosilane. The organic molecules could be anchored onto the mesoporous silica surface through 'direct silanation' mechanism, but the population of the organic groups would be limited to the number of surface silanols, resulting in a low surface coverage (only 22% of full surface coverage was observed when MSNs reacted with vinyl-trichlorsilan). A proper amount of physically absorbed water is of utmost importance to build the functionalized monolyers on MSNs. This absorbed water provides a hydrated interface to hydrolyze organosilanes, which is one of the critical first steps in the deposition of the monolyers. It was found that only two monolayers worth of water (based on the available surface area) is enough to hydrolyze organosilanes and deposition of functional monolayer. However, excess of water should be avoided because the presence of free water would lead to polymerization of organosilanes in a solution.

The use of trichlorosilanes as functional molecules is necessary for the preparation of SAM-MSNs with high surface coverage. A low surface coverage was obtained (about 20%) when trialkoxyorganosilanes were used for deposition of monolayers on the MSNs surface, whereas a high surface coverage was possible when trialkoxyorganosilanes was used to functionalize the bulk mesoporous silica. The reason for this different behavior might be that the functional molecules are very easy to be transferred out of the pores of MSNs during the functionalization process. So, a functional molecule with a higher activity is essential to be hydrolyzed and deposited on the pore walls of MSNs. Trichlorosilanes show higher active toward the absorbed water than trialkoxyorganosilanes. Especially, the hydrolysis of trichlorosilanes generates HCl which can greatly enhance the condensation reaction between neighboring silanes, as well as between the hydroxylsilanes and the polar oxide surface,^24^ leading to closely packed monolayers on the oxide surface.

The stereochemistry of organosilane is another element that significantly affects the surface coverage of the organic functionalized monolayers on SMNs. The vinyl and phenyl-based organosilanes with a short alkane chain generated SAM-MSNs with high surface coverage (100% and 80%, respectively), whereas a relatively low surface coverage (approximately 65%) was obtained when the propyl-based organosilane with a longer alkane chain was used. Isobutyl-trichlorosilane generated the lowest surface coverage (37%). The reason might be that the side methyl groups blocked the condensation reaction between neighboring silanes, resulting in less functional molecules deposition on the MSN surface.

## Conclusions

The SAM-MSNs were synthesized, and the relative surface coverage of the organic monolayers can reach up to 100%. It was found that an appropriate level interfacial hydration, the use of trichlorosilanes as functional molecules, and the functional groups with less steric hindrance are essential to generate MSNs with high surface coverage of monolayers. The functional monolayers provide special attributes (such as binding sites, stereochemical configuration, and special interface properties) for mesoporous silica surface. We believed that the combination of unique structure of MSNs and functionalized monolayers can extend the use of MSNs to a wide array of applications (such as adsorption, sensing, catalysis, and drug delivery) and also benefit the development of new generation of sophisticated functional nanocomposites.

## Abbreviations

BET: Brunauer-Emmett-Teller; BJH: Barrett-Joyner-Halenda; CTAB: cetyltrimethylammonium bromide; FTIR: Fourier transform infrared spectroscopy; Is-SAM-MSNs: isobutyl-functionalized MSNs; MSNs: mesoporous silica nanoparticles; Ph-SAM-MSNs: phenyl-functionalized MSNs; Pr-SAM-MSNs: propyl-functionalized MSNs; SAM-MSNs: self-assembled monolayer functionalize MSNs; TEM: transmission electron microscope; TEOS: tetraethyl orthosilicate; Vi-SAM-MSNs: vinyl-functionalized MSNs; XRD: X-ray diffraction pattern.

## Competing interests

The authors declare that they have no competing interests.

## Authors’ contributions

LMW designed the experiment and wrote the manuscript. LMW, DWS, and PYY carried out experiments. ZHZ helped analyze the characterization results. JW, JZ, LYL, CXC, and YFZ characterized the samples and analyzed the data. All authors read and approved the final manuscript.

## Authors’ information

LMW is currently an associated professor at Shanghai Jiaotong University, China. His research interests include preparation of carbon-based nanomaterilas and their use for in sensors and energy storage. DWS received her B.Sc. degree in the School of Materials Science and Engineering from Shanghai Jiao Tong University (China) and is currently doing her Graduate thesis in Prof Zhang's group in Research Institute of Micro/Nano Science and Technology, Shanghai Jiao Tong University. Her research focus includes carbon nanomaterials for application in sensors and energy storage. ZHZ received his PhD degree in the Shanghai Jiao Tong University. He is now an assistant professor at the University of Electronic Science and Technology of China. His research focus includes nanomaterials for application in solar cells and energy storage. We are grateful for the financial support from the National Natural Science Foundation of China (No. 51272155), National High-Tech R & D Program of China (863, No. 2011AA050504), and the Analytical and Testing Center of SJTU. His research focus now includes the preparation of CNT and CNT field emission display. LYL is an assistant professor at Shanghai Jiao Tong University. Her research focus is characterization of nanomaterials. CXC is currently an associated professor at Shanghai Jiaotong University, China. His research interests include preparation of carbon-based nanomaterilas and their use for in sensors and energy storage. YFZ is currently a professor at Shanghai Jiaotong University, China. His research interests include synthesis of gas-sensing nanomaterials and their applications in nanodevice.
